# Post-Bypass Dexmedetomidine Use and Postoperative Acute Kidney Injury in Patients Undergoing Cardiac Surgery with Cardiopulmonary Bypass

**DOI:** 10.1371/journal.pone.0077446

**Published:** 2013-10-10

**Authors:** Fuhai Ji, Zhongmin Li, J. Nilas Young, Artin Yeranossian, Hong Liu

**Affiliations:** 1 Department of Anesthesiology, First Affiliated Hospital of Soochow University, Suzhou, Jiangsu, China; 2 Department of Anesthesiology and Pain Medicine, University of California Davis Health System, Sacramento, California, United States of America; 3 Department of Internal Medicine, University of California Davis Health System, Sacramento, California, United States of America; 4 Division of Cardiothoracic Surgery, University of California Davis Health System, Sacramento, California, United States of America; Thomas Jefferson University, United States of America

## Abstract

**Background:**

**And Objectives**: The aim of this retrospective investigation was to study the relationships among chronic kidney disease, acute kidney injury (AKI), and potential benefits by post-bypass dexmedetomidine use in patients undergoing cardiac surgery.

**Methods:**

The patient data were reviewed from the institutional Society of Thoracic Surgeons National Adult Cardiac Surgery Database after IRB approval. 1,133 patients were identified and divided into two groups: those who received dexmedetomidine or those who did not during the post-bypass period. The postoperative outcomes include the incidence of AKI, any complication and all cause of mortality.

**Results:**

Post-bypass dexmedetomidine use was associated with significantly reduced the incidence of total AKI (26.1% vs. 33.75%; adjusted OR, 0.7033; 95%CI, 0.540 to 0.916; p=0.0089). In addition, post-bypass dexmedetomidine use was more likely to reduce the incidence of AKI in these patients with preoperative normal kidney function (Stage1; 32.8% to 22.8%; p=0.0233) and mild CKD (Stage 2; 32.8% to 24.7; p=0.0003) after cardiac surgery. Post-bypass infusion of dexmedetomidine was associated with significantly reduced incidence of any complication and 30-day mortalities.

**Conclusions:**

Post-bypass dexmedetomidine use is associated with a significant reduction in the incidence of AKI, especially mild AKI in patients with preoperative normal renal function and mild CKD undergoing cardiac surgery.

## Introduction

Acute kidney injury (AKI) is a common complication after cardiac surgery and associates with adverse outcomes and high healthcare costs [1]. Depending on the preoperative kidney function status, the rate of postoperative AKI can be as high as 30% in cardiac surgery patients. AKI is associated with up to 60% mortality rates of all cardiac surgery patients and a 25-fold increase in mortality following cardiac valve surgeries [2,3]. The pathogenesis of AKI is multifactorial and involves hemodynamic, inflammatory and ischemia/reperfusion (I/R) injury [4]. It is well known that renal function is closely associated with hemodynamic and sympathetic nervous system activity.

 Since cardiac surgery triggers endocrine responses that stimulates the hypothalamus-pituitary-adrenal axis, the sympathetic nervous system, resulted in epinephrine and norepinephrine release and caused an unstable hemodynamics that is detrimental to renal function [5]. It has been reported that peak intraoperative plasma concentrations of norepinephrine and epinephrine occurred after cardiopulmonary bypass (CPB) [6]. This is a critical period with a higher blood catecholamine level that is detrimental to patients [7]. Studies demonstrated that the hemodynamic stabilizing and sympatholytic effects produced by clonidine, an alpha-2 agonist, could prevent the deterioration of renal function after cardiac surgery [8,9]. This may be also associated with some potentially renal-protective effects including inhibition of rennin release, increased glomerular filtration and increased secretion of sodium and water produced by alpha2-adrenoceptor activation [10]. Dexmedetomidine is a highly selective, shorter-acting intravenous alpha-2 agonist with an alpha-2 to alpha-1 selectivity ratio of 1600:1 [11]. Study also found that dexmedetomidine could effectively abolish the increase of sympathetic activation and vasoconstriction induced by cocaine [12]. By stabilizing the sympathetic system, exerting anti-inflammatory effects and attenuating I/R injury, dexmedetomidine has been shown to protect renal function in laboratory studies [13,14]. However, no study has demonstrated the benefit of dexmedetomidine on renal function in cardiac surgery. Leino and colleagues reported that use of intravenous dexmedetomidine did not alter renal function in a cohort of relatively low-risk elective coronary artery bypass graft (CABG) patients but was associated with an increase in urinary output, but the relationship among post-bypass dexmedetomidine use, preoperative renal function and postoperative AKI were not studied [15]. Thus, this study was designed to investigate the relationships among preoperative renal function, chronic kidney disease (CKD), AKI and outcomes, and potential benefits by post-bypass dexmedetomidine administration in patients undergoing cardiac surgery with cardiopulmonary bypass.

## Materials and Methods

### Study Design

**Figure 1:**
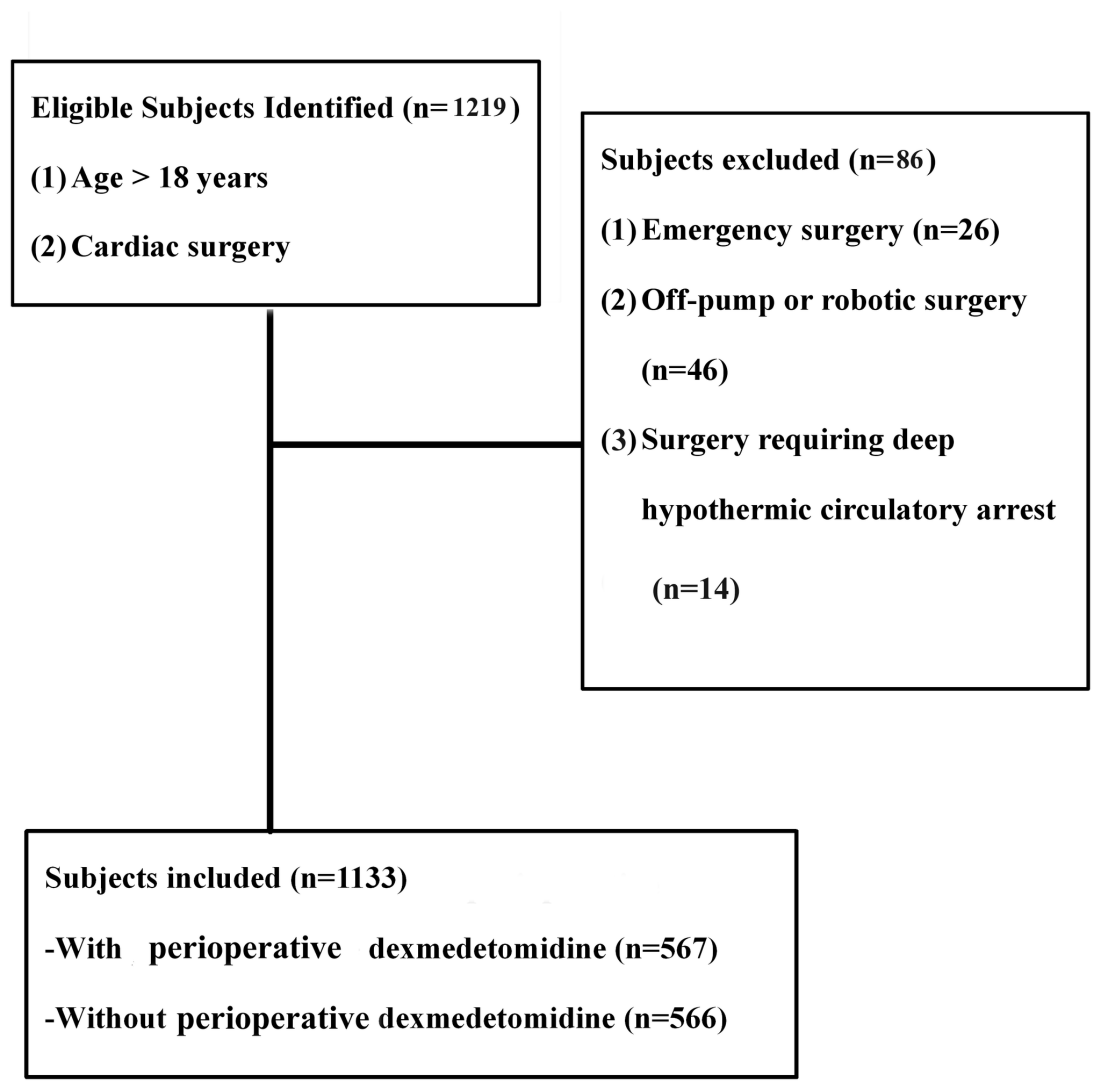
Recruiting of study sample.

This study was a retrospective cohort study involving 1,219 consecutive cardiac surgery (CABG and/or valve surgery, congenital cardiac surgery and aortic surgery) patients in a single tertiary medical center (University of California Davis Health System) from January 1, 2006 to December 31, 2011. The study was reviewed and approved by the University of California Davis Institutional Review Board. Due to the nature of the retrospective study, the written consent was not given by the patients for their information stored in the hospital database to be used for research. This waive of consent was approved by the IRB. Patients underwent emergency surgery, off-pump or robotic surgeries and surgeries requiring deep hypothermic circulatory arrest were excluded from this study (Figure 1). 1,133 patients were identified and divided into two groups: those who received dexmedetomidine (DEX group, n=567, 50.04%) or those who did not receive dexmedetomidine (Non-DEX group, n=566, 49.96%) during the post-bypass period (Figure 1). This study is registered at http://www.clinicaltrials.gov/ct2/show/NCT01683448?term=NCT01683448&rank=1 and the identifier is: NCT01683448. 

### Data Collection

The patient data were collected and reviewed from the institutional Society of Thoracic Surgeons (STS) National Adult Cardiac Surgery Database and the hospital medical records that included demographics, patient history, medical record information, preoperative risk factors, preoperative medications, intraoperative data, renal failure, in-hospital and 30-day all cause mortality. Independent investigators prospectively collected the data on each patient during the course of the hospitalization. Post-bypass dexmedetomidine use was defined as an intravenous infusion (0.24 to 0.6mcg/kg/hr) initiated after CPB and continued for less than 24 hours postoperatively in the ICU. Infusion rate of dexmedetomidine was adjusted according to patients’ hemodynamic parameters. The decision on which patients received dexmedetomidine was at the discretion of the attending anesthesiologists who provided anesthesia care to the patients.

### Major and secondary outcomes

Major outcomes of this study were postoperative AKI, in-hospital and 30-day all cause of mortalities. Secondary outcomes included postoperative length of mechanical ventilation, postoperative renal failure (RF), length of intensive care unit (ICU) stay, length of hospital stay (LOS) and 30-day readmission. Based on the STS criteria, the following definitions were used: In-hospital mortality: whether the patient has been declared dead within this hospital admission. This includes all causes of death, including those causes clearly unrelated to the operation. 30-day mortality: whether the patient was alive or dead at 30 days post surgery (whether in hospital or not). The primary method used to verify the patient's mortality status were phone call to patient or family; letter from medical providers; evidence of life in medical record (laboratory tests, cardiac rehabilitation visits, etc.); office visits to surgeon after discharge; social security death master file. Postoperative RF: acute or worsening RF resulting in one or more of the following: increase in serum creatinine >2.0 mg/dL or two-fold increase of most recent preoperative serum creatinine or a new requirement for dialysis, and 30-day readmission is defined as the patient was readmitted as an in-patient within 30 days from the date of initial surgery for any reason. This includes readmissions to acute care, primary care institutions only, not to rehabilitation hospitals or nursing homes. http://www.sts.org/documents/pdf/trainingmanuals/adult2.73/V-c-AdultCVDataSpecifications2.73.pdf (accessed at June 30, 2012) 

The patient baseline kidney functions were divided into five stages based on preoperative estimated glomerular filtration rate (eGFR ml/min/1.73m^2^): Stage 1, normal eGFR (>90); Stage 2, mild decreased eGFR (60-89); Stage 3, moderate decreased eGFR (30-59); Stage 4, severe decreased eGFR (15-29); Stage 5, on dialysis or kidney failure (eGFR <15) [16,17]. eGFR was calculated based on Modification of Diet in Renal Disease equations [17]. Postoperatively, AKI was divided into three stages based on Acute Kidney Injury Network (AKIN) criteria [18]: increase in serum creatinine above baseline: ≥ 1.5 to 2 fold or creatinine increase ≥0.3mg/dL (Stage 1), >2 to 3 fold (Stage 2), >3 fold or creatinine increase > 4.0 mg/dL or acute increase >0.5mg/dL (Stage 3).

### Statistical Analysis

Continuous and categorical variables were reported as mean ± SD or percentages, and compared with a 2-sample *t* tests or a chi-square test (two tailed), respectively. Univariate and multivariate logistic regression were performed to assess associations between dexmedetomidine use and demographic, therapeutic and clinical outcome variables. To mitigate selection bias in dexmedetomidine use, we computed the propensity score, that is, the conditional probability of each patient receiving dexmedetomidine with a multivariable logistic regression model that includes patient demographic and clinical risk factors (Figure 1). We presented parsimonious models with a backward selection method from all candidate risk factors (α=0.1). The candidate risk factors were selected on the basis of the literature reviews, clinical plausibility, and variables collected in the database. The candidate independent variables included demographic and clinical risk factors in Table 1. The parsimonious multivariable propensity model for dexmedetomidine use included status of procedure, preoperative family of coronary arterial disease (CAD), preoperative hypercholesterolemia, preoperative dyslipidemia, preoperative congestive heart failure (CHF), preoperative ejection fraction (EF), preoperative beta-blockers, preoperative lipid lowing medications and perfusion time (Table 2). Bivariate logistic regression analysis was initially performed to identify significant predictors of postoperative AKI and a propensity-weighted logistic regression model was used for risk-adjustment for postoperative AKI in which an inverse (estimated) propensity score as weights for patients with dexmedetomidine and the inverse of 1 minus the propensity score for patients without dexmedetomidine. The model included patient preoperative risk factors and use of dexmedetomidine as an independent factor. All models fit analysis was evaluated with the Hosmer-Lemeshow goodness-of-fit statistic. The C statistic was reported as a measure of predictive power. Based on the propensity of dexmedetomidine use we classified all patients into quintile where quintile 1 contained patients with lowest propensity scores and quintile 5 contained patients with the highest propensity scores. Then, with a general linear model, we compared the propensity weighted and risk adjusted postoperative AKI between the cohort of dexmedetomidine used and the cohort of no dexmedetomidine used for each propensity-matched quintile. We also classified all patients into 5 stratifications according to the stage of preoperative CKD and compared the predicted postoperative AKI between dexmedetomidine use and no dexmedetomidine use cohorts for each preoperative CKD stages. All differences in statistical analysis were considered significant if p <0.05. All data analyses were conducted with SAS version 9.3 (Cary, NC).

**Table 1:** Demographic and Clinical Characteristics.

**Characteristics**	**Dexmedetomidine**		**P value**
	Yes (n=567)	No (n=566)	
Age (>65)	254(44.97)	250(44.17)	0.9002
Gender (M)	405(71.6)	401(70.85)	0.7785
BMI	29.40 (6.38)	29.87 (7.1)	0.2328
Race (non-white）	184(32.45)	182(32.33)	0.9658
Status (urgent）	269(47.44)	358(63.25)	<0.0001
Smoking	298(52.56)	312(55.12)	0.3865
Current Smoking	489(18.87)	476(22.26)	0.1582
**Chronic lung disease**			
None	489(86.24)	476(84.1)	0.7190
Mild	55(9.70	73(12.9)	
Moderate	18(3.17)	5(2.12)	
Severe	5(0.88)	5(0.88)	
Cerebrovascular Disease	94(16.58)	111(19.61)	0.1851
Peripheral Vascular Disease	80(14.11)	84(14.84)	0.7265
Family History of CAD	109(19.22)	168(29.68)	<0.0001
Diabetes	203(35.8)	177(31.27)	0.1065
Hypertension	436(76.9)	436(77.03)	0.9567
Hypercholesterolemia	394(69.49)	461(81.45)	<0.0001
Dyslipidemia	383(67.55)	253(44.7)	<0.0001
Preoperative MI	185(32.63)	246(43.46)	0.0002
CHF	179(31.57)	42(7.42)	<0.0001
IABP Use	38(6.7)	50(8.83)	<0.0001
EF %	52.45 (12.82)	49.68 (13.60)	0.0004
**Preoperative medications**			
ACEI	284(50.09)	326(57.6)	0.0113
Beta Blockers	394(69.49)	374(66.08)	0.2195
ADP Inhibitors	27(4.76)	65(11.48)	<0.0001
Nitrates	16(2.82)	20(3.53)	0.4949
Anticoagulants	103(18.17)	146(25.8)	0.0019
Antiplatelet	9(1.59)	6(1.06)	0.4378
Coumadin	42(7.41)	37(6.54)	0.5654
Inotropes	2(0.35)	7(1.24)	0.0939
Steroids	18(3.17)	19(3.36)	0.8630
Aspirin	438(77.25)	456(80.57)	0.1714
Lipid Lowering	371(65.43)	296(52.3)	<0.0001
GPIIb/IIIa Inhibitor	15(2.65)	33(5.83)	0.0078
**Procedural characteristics**			
Type of Surgery			
CABG only	304(53.7)	330(58.30)	0.1183
CABG +Valve	118(20.77)	153(27.03)	0.0135
CABG +other	116(5.11)	39(6.89)	0.2058
Valve+(Valve +other)	116(20.42)	44(7.77)	<0.0001
Vessels of CABG (>4)	94(16.58)	133(23.49)	0.0035
Perfusion Time (min)	181.20 (75.47)	199.80 (81.60)	<0.0001
Cross Clamp Time (min)	128.90 (63.97)	144.80 (62.47)	<0.0001
**Propensity Score**	0.61 (0.21)	0.39 (0.20)	<0.0001

Values are n (%) for categorical variables and mean±SD for continuous variables. BMI, body mass index; CAD, coronary arterial disease; CKD, chronic kidney diseases; e-GFR, estimated glomerular filtration rate; MI, myocardial infarction (MI); CHF, chronic heart failure; IABP, intra-aortic balloon pump; EF, ejection fraction; ACEI, angiotensin converting enzyme inhibitors; ADP, adenosine diphosphate; GPIIb/IIIa, glycoprotein IIb/IIIa; CABG, coronary arterial bypass graft

**Table 2:** Multivariable Propensity Model for Dexmedetomidine Use

**Risk factors**	**Adjusted OR**	**95%CI**	**P valve**
Status (Urgent vs. Elective)	0.578	0.441-0.758	<0.0001
Family History of CAD	0.689	0.504-0.943	0.0199
Pre-op Hypercholesterolemia	0.507	0.337-0.762	0.0011
Pre-op Dyslipidemia	2.969	2.164-4.073	<0.0001
Pre-op CHF	4.866	3.248-7.29	<0.0001
Pre-op EF	1.015	1.004-1.026	0.0066
Pre-op Beta Blockers	1.509	1.128-2.018	0.0056
Pre-op Lipid Lowering	0.611	0.446-0.836	0.0021
Perfusion Time	0.997	0.995-0.999	0.0005

OR, odds ratio; CI, confidence interval; CAD, coronary artery disease; Pre-op, preoperative; CHF, congestive heart failure; CABG, coronary artery bypass graft; EF, ejection fraction

## Results

Preoperative demographic and clinical data of the patients who did and did not receive post-bypass dexmedetomidine are presented in Table 1. Age (>65 years) and gender (male) distributions were similar between groups as was body mass index (BMI), race (non-white), smoking, last creatinine level and dialysis, percentage of patients with chronic lung disease, cerebrovascular disease, peripheral vascular disease, diabetes, hypertension and CKD. Patients receiving post-bypass dexmedetomidine use were more likely to have prior CHF, history of RF, dyslipidemia and a higher EF value whereas patients in Non-DEX group were more likely to have family history of CAD, hypercholesterolemia, preoperative myocardial infarction and a larger percentage of patients receiving intra-aortic balloon pump (IABP). In addition, patients receiving post-bypass dexmedetomidine were more likely to receive preoperative lipid lowering medications, whereas there were a larger percentage of patients receiving preoperative angiotensin converting enzyme inhibitors, adenosine diphosphate receptor inhibitors, anticoagulants and glycoprotein IIb/IIIa Inhibitors in the Non-DEX group. The uses of preoperative beta-blocker, nitrates, antiplatelet agents, Coumadin, inotropes, steroids and aspirin were similar between the two groups. 

Procedural characteristics, the distributions of surgeries including CABG and CABG combined with other procedure were similar in both groups. However, there were more patients received valve surgery or combined with other procedures in DEX group, whereas there were more patient received CABG surgery in Non-DEX group. In addition, perfusion time and aortic cross-clamp time were longer in Non-DEX group (Table 1).

Before surgery, there were 214 patients in Stage 1, 573 in Stage 2, 294 in Stage 3, 24 in Stage 4 and 28 patients in Stage 5. CKD is defined as eGFR <60 ml/min/1.73m^2^ presented in 30.54% of this cohort of patients undergoing cardiac surgery [16]. The values of eGFR, preoperative creatinine level and the distribution of CKD stages were similar in two groups (Table 3). 

**Table 3:** Preoperative Kidney Function Characteristics.

**Characteristics**	**Dexmedetomidine**		**P value**
	Yes (n=567)	No (n=566)	
CKD Stage			
1	114(20.11)	100(17.67)	0.2947
2	271(47.8)	302(53.36)	0.0613
3	149(26.28)	145(25.62)	0.8
4	16(2.82)	8(1.41)	0.0999
5	17(3)	11(1.94)	0.2531
eGFR	71.70 (28.98)	71.49 (23.89)	0.8983
Last creatinine level	1.24 (1.09)	1.13(0.68)	0.0625
History of Renal Failure	31(5.46)	14(2.47)	0.0101
Dialysis	19 (3.35)	11(1.94)	0.1416

Values are n (%) for categorical variables and mean±SD for continuous variables. CKD, chronic kidney diseases; eGFR, estimated glomerular filtration rate

**Figure 2:**
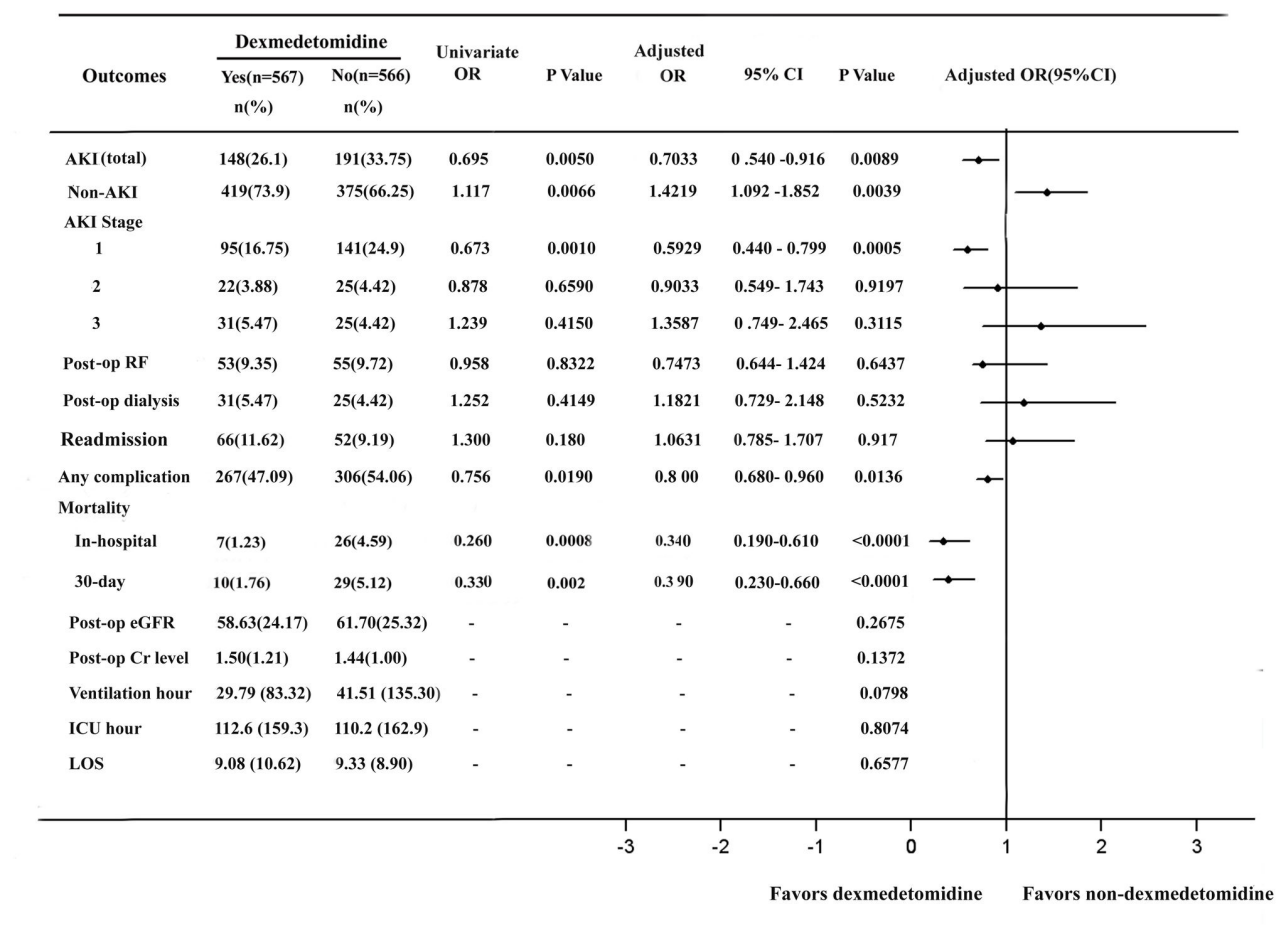
Effects of dexmedetomidine on postoperative AKI and other outcomes in patients undergoing cardiac surgery. Values are numbers (%) for categorical variables and mean± SD for continuous variables. OR, odd ratio; CI, confidence interval; AKIN, acute kidney injury network; AKI, acute kidney injury; RF, renal failure; Post-op, postoperative; eGFR, estimated glomerular filtration rate.

Overall, the incidence of AKI was closely correlated with the baseline renal function, the more advanced CKD at the baseline, the more frequent the postoperative AKI occurred (ranged from 27.8% if eGFR ≥90 ml/min/1.73m^2^ to 73.2% if eGFR at 15-30 ml/min/1.73m^2^) (Table 4). Post-bypass dexmedetomidine use was associated with significantly reduced the incidence of total AKI (26.1% vs. 33.75%; Adjusted OR, 0.7033; 95%CI, 0.540 to 0.916; p=0.0089). Moreover, dexmedetomidine use was more likely to reduce the incidence of mild AKI (Stage 1; 16.75% vs. 24.9%; Adjusted OR, 0.5929; 95%CI, 0.440 to 0.799; p=0.0005) (Figure 2). In addition, post-bypass dexmedetomidine use was more likely to reduce the incidence of postoperative AKI in these patients with normal preoperative kidney functions (Stage 1: 32.8% to 22.8%; p=0.0233) and mild CKD (Stage 2: 32.8% to 24.7%; p=0.0003) after cardiac surgery whereas there were no statistical differences in the incidence of postoperative AKI in these patients with preoperative moderate (Stage 3: 32.2% vs. 37.0%; p=0.6738), severe CKD (stage 4: 64.0% vs. 80.8%; p=0.7315) and renal failure or on dialysis (Stage 5: 63.6% vs. 77.9%; p=0.6416) (Table 4). 

**Table 4:** Predicted Acute Kidney Injury by Preoperative Chronic Kidney Disease Stage

**CKD Stage**	**Mean Predicted Post-AKI**			**95% Confidence Intervals**		**P valve**
	**DEX**	**Non-DEX**		**DEX**	**Non-DEX**	
1	0.228	0.328		0.1886-0.268	0.286-0.371	0.0233
2	0.247	0.328		0.222-0.273	0.304-0.352	0.0003
3	0.322	0.370		0.288-0.357	0.335-0.405	0.6738
4	0.640	0.808		0.534-0.745	0.659-0.957	0.7315
5	0.636	0.779		0.533-0.738	0.652-0.907	0.6416

Data stratified comparison in predicted postoperative AKI by preoperative chronic kidney disease stage and adjusted by propensity score. CKD, chronic kidney disease; AKI, acute kidney injury; DEX, dexmedetomidine

The quintile of propensity score reveled that patients who received dexmedetomidine in all five groups of quintile were significantly lower with respect to postoperative AKI when compared to the patients in the Non-DEX group (9.78% vs.21.85%, p=0. 0451; 20.44% vs. 36.13%, p<0.0001; 24.45% vs. 40.70%, p<0.0001; 25.31% vs. 43.93%, p<0.0001; 38.39% vs. 67.71%, p<0.0001; respectively) (Table 5). 

**Table 5:** Predicted Postoperative Acute Kidney Injury by Quintile

**Quintile**	**Number**		**Mean propensity score**		**p valve**	**Mean predicted postoperative AKI**		**p valve**
	DEX	Non-DEX	DEX	Non-DEX		DEX	Non-DEX	
1	33	193	0.194	0.179	0.8163	0.09781	0.2185	0.0451
2	82	145	0.352	0.346	0.9981	0.2044	0.3613	<0.0001
3	108	119	0.489	0.49	1	0.2545	0.407	<0.0001
4	159	68	0.644	0.624	0.1536	0.2531	0.4393	<0.0001
5	185	41	0.846	0.844	1	0.3839	0.6771	<0.0001

Data stratified comparison in predicted postoperative AKI by Quintile of propensity score. AKI, acute kidney injury; DEX, dexmedetomidine

Post-bypass dexmedetomidine use was associated with significantly reduced in the incidence of any complication (47.09% vs. 54.06%, adjusted OR, 0.800; p= 0.0136), in-hospital (1.23% vs. 4.59%; adjusted OR, 0.340; p<0.0001) and 30-day (1.76% vs. 5.12%; adjusted OR, 0.390; p<0.0001) mortalities. However, there were no statistical differences in 30-day readmission, ventilation hour, ICU hour and LOS (Figure 2). 

**Figure 3:**
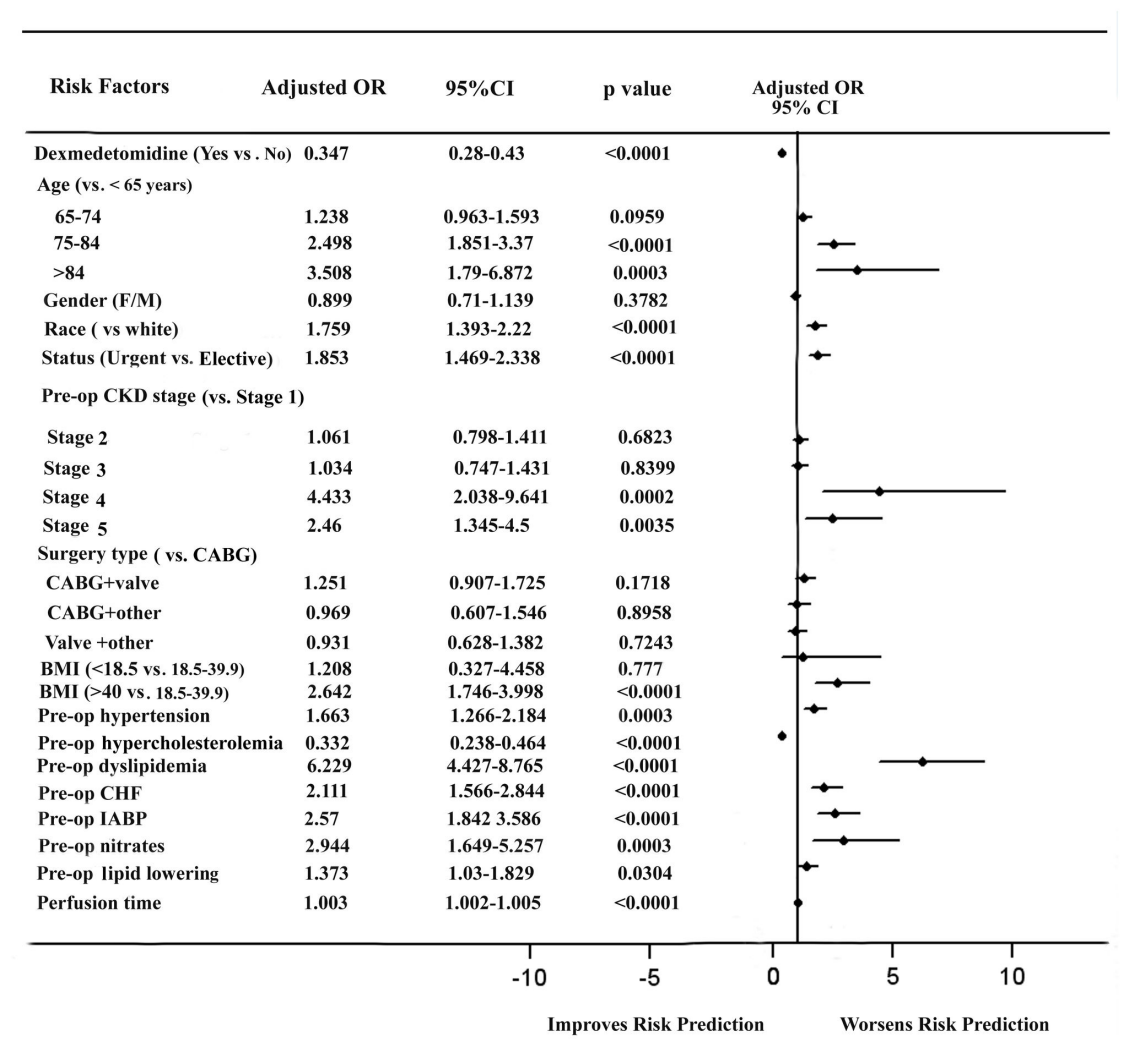
Propensity adjusted risk model for postoperative AKI following cardiac surgery. Values are n (%) for categorical variables and mean± SD for continuous variables. OR, odd ratio; CI, confidence interval; CKD, chronic kidney disease; CABG, coronary artery bypass graft; BMI, body mass index; Pre-op, preoperative; CHF, congestive heart failure; IABP, intra-aortic balloon pumping.

After adjusted for propensity scores and covariates, multivariate logistic regression model analysis revealed that elderly patients (>75 years), non-white race, urgent surgery, severe CKD (Stage 4 and 5), obesity (BMI>40), preoperative hypertension, dyslipidemia, CHF, IABP use, nitrates, lipid lowering medications and perfusion time significantly increased the risk of postoperative AKI. Conversely, post-bypass use of dexmedetomidine was found to have a significant benefit in protecting patients against AKI (OR, 0.347; 95%CI, 0.28 to 0.43; p<0.0001) (Figure 3).

## Discussion

This was the first study to demonstrate that 1) patients undergoing cardiac surgery are often concomitant with preoperative CKD (30.54%); 2) baseline renal function or eGFR is associated with postoperative AKI which the probability increased proportionally with the worsening of preoperative kidney function; 3) post-bypass dexmedetomidine use was associated with a decrease in postoperative AKI, particularly in patients with normal preoperative kidney functions and mild CKD; 4) post-bypass dexmedetomidine use was also associated with a decrease in postoperative in-hospital and 30-day mortalities and the incidence of any complication. 

AKI previously referred to as acute renal failure (ARF) with an estimated incidence as high as 30 % in cardiac surgery patients. Patients suffered from AKI had significant poor outcomes including increased short-term and long-term mobility and mortality, prolonged hospital stay that further translates into higher costs [1,2,19]. Studies in cardiac surgery patients have demonstrated that AKI added additional 1–5 % to the in-hospital mortality and added up to 24% in patients who require acute dialysis [20]. The pathogenesis of AKI is complex and multifactorial. It has been suggested that activation of sympathetic nervous system, I/R injury, systemic inflammatory responses, increased metabolic products, neurohormonal activation and oxidative stress are among the potential causes [21].

Certain predisposing factors of AKI existed in most patients underwent CPB [22]. Our results indicated that several risk factors included older age, non-white race, urgent surgery, preoperative kidney diseases, obesity, preoperative hypertension, dyslipidemia, CHF, IABP use, nitrates, lipid lowering agents and longer perfusion time all contributed to increase postoperative AKI in patients undergoing cardiac surgery. Our results also demonstrated that postoperative AKI was associated with the degree of preoperative CKD. Patients with severe CKD were more likely to develop postoperative AKI. Decreased renal function reserve, pre-existing renovascular disease and impairment of kidney auto-regulation could be the contributing factors for the postoperative renal function deterioration. These hemodynamic perturbations during CPB make the kidneys with existing CKD even more vulnerable to any further ischemic or other insults [23,24].

Dexmedetomidine is a novel α_2_ adrenoceptor agonist with the property of sedation, analgesia, inhibition of central sympathetic outflow, hemodynamic stabilization, anti-inflammation and diuresis [5,11,15]. It is a highly selective, shorter-acting intravenous alpha-2 agonist with a remarkable binding specificity for the α_2_ adrenoceptor [11]. Dexmedetomidine has been shown to protect heart, brain, kidneys and lungs in laboratory studies [12,25-27]. Study has suggested that α_2_ adrenoceptors exist widely in the renal peri-tubular vasculatures, proximal and distal tubules [28]. Clinical study demonstrated that α_2_ adrenoceptor agonists enhance urine flow and perioperative renal function in non-cardiac surgery patients [29]. Pretreatment with clonidine has shown beneficial effects on renal function after cardiac surgery [8]. However, study has not found significant difference in postoperative creatinine clearance even with an increase in urinary output with dexmedetomidine [15]. Our result further pointed out dexmedetomidine was more likely to protect kidneys against mild AKI after cardiac surgery. Furthermore, we also found post-bypass dexmedetomidine use was associated with reduced postoperative AKI in patients with normal kidney function and mild CKD. These findings are clinically significant because even a mild impairment in renal function is associated with increased mortality following cardiac surgery [19]. The mechanisms of dexmedetomidine on kidney function remain unknown. It is conceivable that dexmedetomidine attenuates surgical stress-induced increases in circulating epinephrine and norepinephrine and maintain renal blood flow and glomerular filtration. Therefore, it can protect the kidney against the adrenergic-mediated vasoconstriction by activation of α-2c receptors in the wall of vascular smooth muscle [8,30,31]. Dexmedetomidine can also exert direct vascular effects in the kidneys by decreasing the sympathetically mediated presynaptic release of norepinephrine in the kidneys, which could promote renal arterial vasodilatation [32]. A few plausible explanations including activating cell survival signal phosphatidylinositol kinase via α_2_ adrenoceptors to reduce cell death and high-mobility group protein B1 release and subsequently inhibits toll-like receptor 4 signaling, activating the cholinergic anti-inflammatory pathway, enhancing macrophage phagocytosis for bacterial clearance and stabilizing hemodynamics [5,13,33,34]. Finally, dexmedetomidine induces diuresis, possibly by suppressing the secretion of arginine vasopressin, may have had a salutary effect on glomerular filtration [35]. It has been reported that dexmedetomidine persevered calculated creatinine clearance and reduce the severity of AKI by preventing tubule obstruction and decreasing oxygen consumption [29,36].

There are several limitations in this investigation. First, it is a retrospective study that has shown a relationship among dexmedetomidine infusion, preoperative CKD and postoperative AKI, but does not establish a cause and effect relationship as prospective study does. Secondly, post-bypass dexmedetomidine infusion may not reduce the incidence of severe renal dysfunction, but mild AKI, which may adversely affect outcome even without preexisting renal dysfunction. Finally, although multivariate regression was used in this study to reduce overt biases, potential biases may still exist which included potential multiple and uncontrollable confounding factors destined for a non-randomized study.

In conclusion, this study is the first to demonstrate that post-bypass dexmedetomidine infusion is associated with a significant decrease in the incidence of mild acute kidney injury in these cardiac surgical patients with preoperative normal renal function and mild chronic kidney disease. The post-bypass use of dexmedetomidine is also associated with a significant decrease in in-hospital, 30-day mortality and the incidence of postoperative overall complications. A prospective, multicenter randomized study focused on the relationship between the use of dexmedetomidine and acute kidney injury in cardiac surgery patients is indicated to confirm these findings.

## Supporting Information

Figure S1 **The distribution of patients’ weight after propensity adjustment. Dex_YN, patients received dexmedetomidine or those who did not; ps, propensity score.** (TIF)
