# Supplementary figures and images for: Post-Bypass Dexmedetomidine Use and Postoperative Acute Kidney Injury in Patients Undergoing Cardiac Surgery with Cardiopulmonary Bypass
Source: PLoS One. 2013 Oct 10;8(10):e77446. doi: 10.1371/journal.pone.0077446 (PMC3795046; doi:10.1371/journal.pone.0077446)

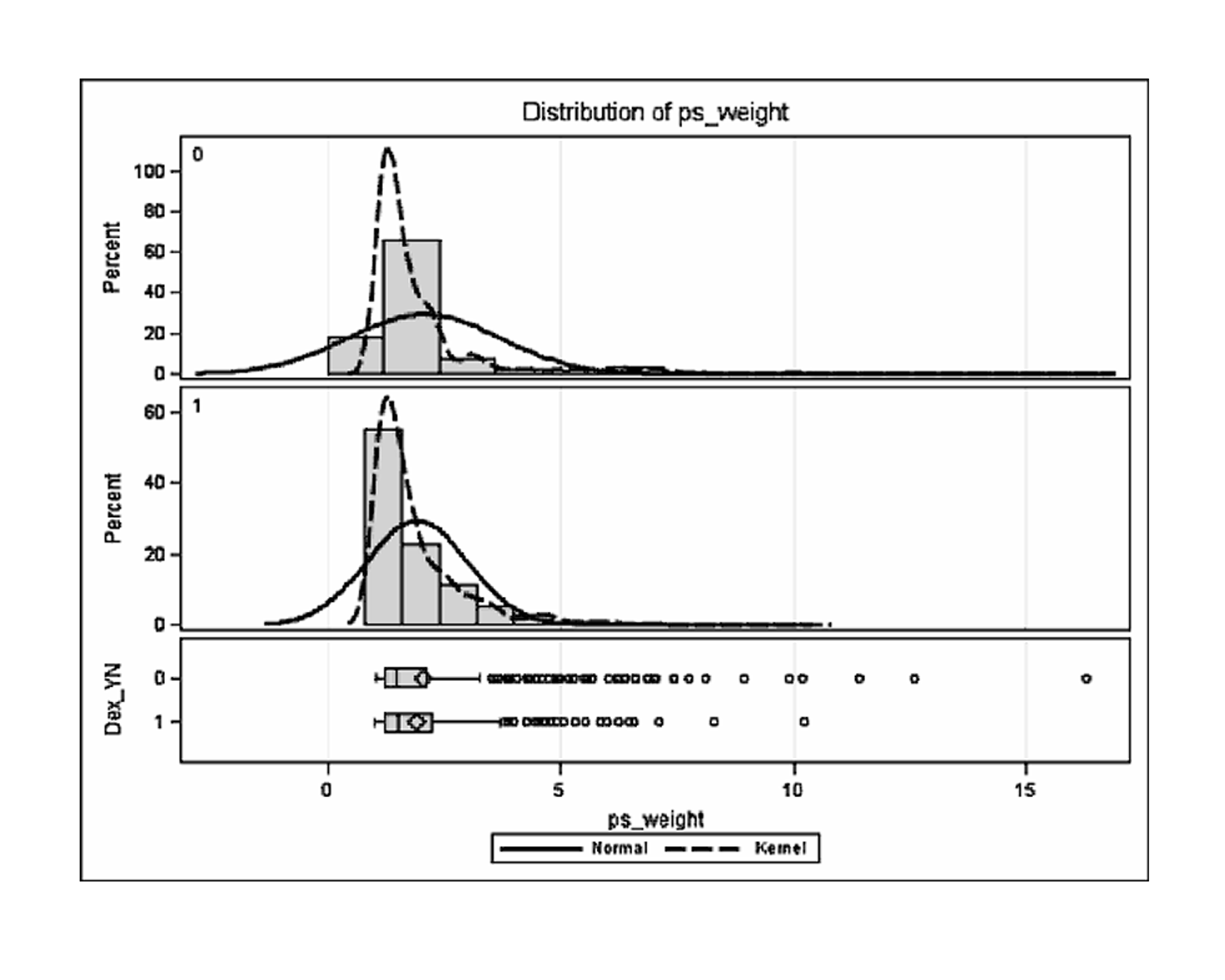

Supplement: Figure S1 — The distribution of patients’ weight after propensity adjustment. Dex_YN, patients received dexmedetomidine or those who did not; ps, propensity score. (TIF) [file pone.0077446.s001.tif]
